# Miocene cladocera from Poland

**DOI:** 10.1038/s41598-020-69024-9

**Published:** 2020-07-21

**Authors:** Henri J. Dumont, Agnieszka Pociecha, Edyta Zawisza, Krystyna Szeroczyńska, Elżbieta Worobiec, Grzegorz Worobiec

**Affiliations:** 1 Department of Biology, Gent University, 9000 Ghent, Belgium; 2 Institute of Nature Conservation, Polish Academy of Sciences, Adama Mickiewicza Av. 33, 31120 Kraków, Poland; 3 Institute of Geological Sciences, Polish Academy of Sciences, Twarda 51/55, 00818 Warsaw, Poland; 4 W. Szafer Institute of Botany, Polish Academy of Sciences, Lubicz 46, 31512 Kraków, Poland

**Keywords:** Ecology, Zoology, Climate sciences, Ecology, Environmental sciences, Limnology

## Abstract

The Bełchatów Lignite Mine of Poland is a treasure-cove for mid-to late Miocene plant and animal fossils, deposited in a slow-flowing river valley with swamps and oxbow lakes. Here, we report the finding of abundant fossil anomopod cladocerans. Some are three-dimensionally preserved, including the taxonomically important trunk limbs. They pertain to the families Chydoridae and Bosminidae, with species similar to but distinct from modern ones. All are members of the zooplankton, though some are littoral while others are pelagic in nature. Morphological stasis in these families is not outspoken as in the Daphniidae and the stasis hypothesis, based on ephippia only, is challenged. The absence of Daphnia is conspicuous and ascribed to a combination of fish predation and local water chemistry. Its place in the oxbow lakes is taken by at least two Bosmina species, one of which is undescribed. We consider this a case of paleo-competitive release. For Bosminidae, these are the first certified fossils predating the Pleistocene.

## Introduction

**Figure 1:**
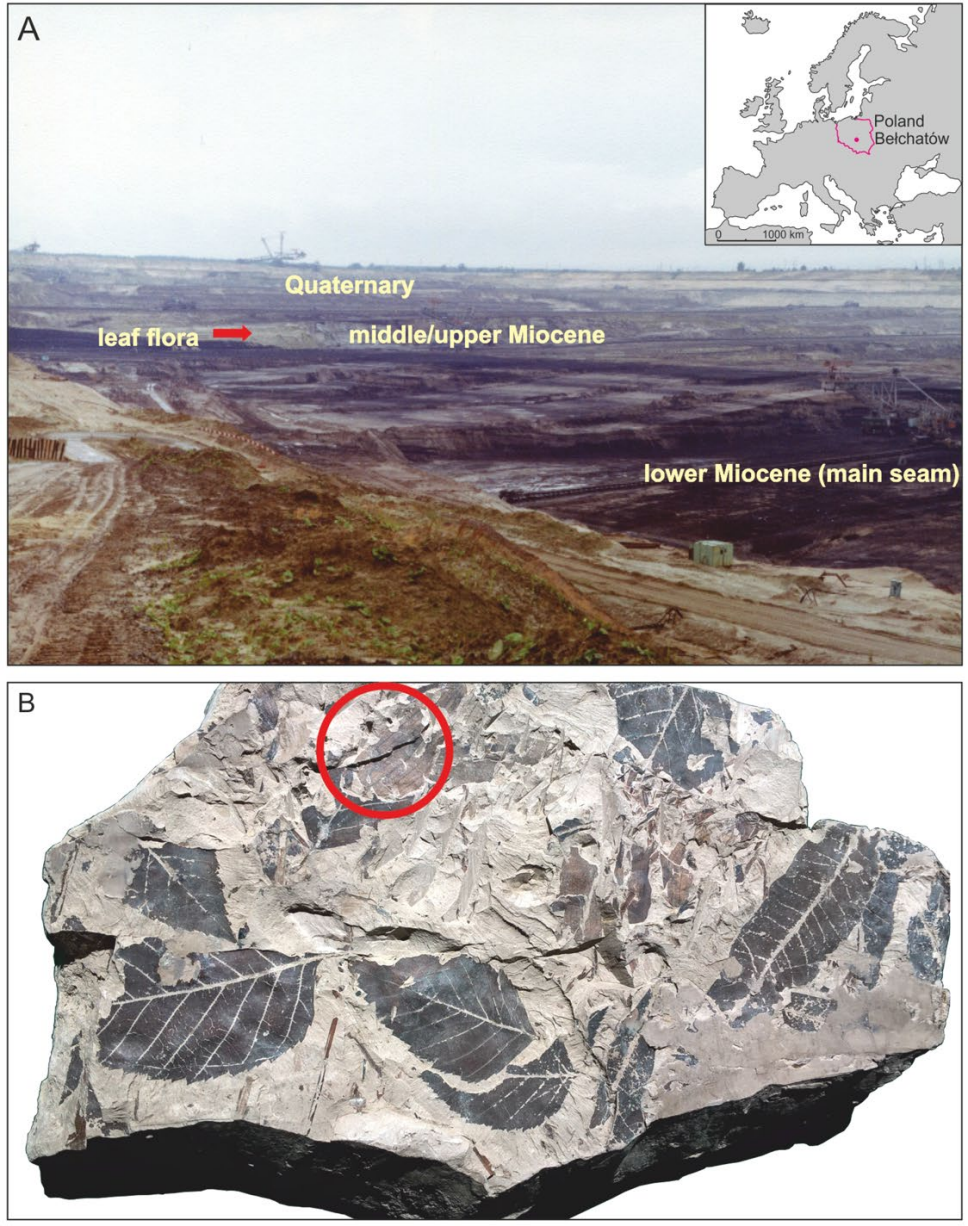
(**A**) View of the Bełchatów Lignite Mine outcrop (photo from the nineties of the twentieth century, when the material was collected). The sedimentary unit with deposits with the cladoceran fossils is to the lower right of the picture (photo: G. Worobiec). (**B**) Piece of mudstone with cladoceran remains (Collection KRAM-P 225, stored in the W. Szafer Institute of Botany, Polish Academy of Sciences). On the exposed surface leaves of trees and shrubs as well as the water plant *Potamogeton* (red circle) are seen (photo: A. Pociecha).

The lignite mine at Bełchatów, Central Poland (Fig. 1), has recently yielded abundant Miocene remains of several species of branchiopod microcrustaceans. They pertain to the order Anomopoda or water fleas, families Chydoridae and Bosminidae. The main synapomorphy of the anomopods is the ephippium, a structure in which sexual or resting eggs are deposited and that forms when external conditions deteriorate. The Daphniidae include the well-known and speciose genus Daphnia, a highly specialized pelagic component of the freshwater zooplankton. The ephippium fossilizes well and is mostly the only structure that survives as a fossil. The Chydoridae are mostly found in the littoral of lakes and ponds, among water plants. The Bosminidae are pelagic, like Daphnia, but are much smaller (around 0.5 mm in body size) while Daphnia and other daphniids may reach up to 6–7 mm.

The fossil-bearing deposits belong to a clayey-sandy (I-P) unit considered to be of mid to late Miocene age^1,2^. Beside by geology, this age is supported by fission track dating, and fossils of different animal (mollusks, fish) and plant (higher plants, water plants and algae) groups^1–7^. The fission track ages were 16.5 to 18.1 million years, while the Miocene, also known as the age of mammals, extended from 23 to 5.3 million years BP.

The Branchiopoda (a class of the Crustacea composed ten extant orders) include some of the most ancient extant crustaceans (the Anostraca or fairy shrimp) with several credible fossils^8^. However, the fossil record is patchy. While the oldest known anostracan-like representatives date back to the Cambrian, fossils of the four extant so-called cladoceran orders, with an estimated 1,000 extant species, is poor^9^ and somewhat paradoxical. The order Ctenopoda is known from ca 60 extant species and several Mesozoic fossils (possibly representing orders in their own right) but is rare in the most abundant source of fossils, the sediments of late Pleistocene–Holocene lakes^10,11^. The Anomopoda, in contrast, have to date a limited Mesozoic fossil record, but their subfossils abound in Holocene lake sediments.

**Figure 2:**
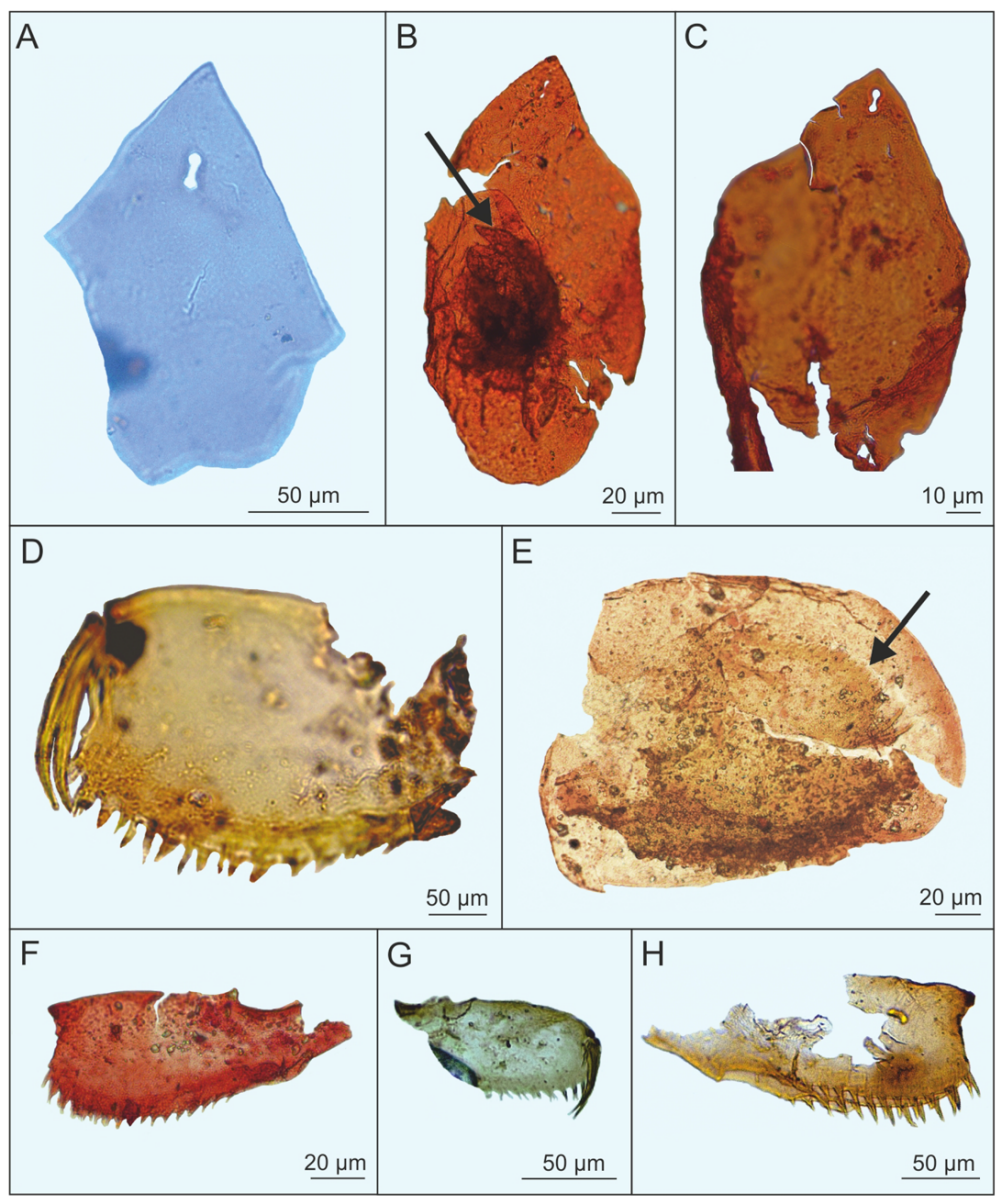
*Alona* with two head pores from Miocene mudstone from Bełchatów [(**A**–**C**), headshields; (**B**) arrow, labrum; (**D**, **F**, **G**, **H**) abdomens; (**E**) shell with abdomen] (photo: A. Pociecha, E. Zawisza).

The family Chydoridae is represented in Bełchatów by at least four genera: *Alona*, *Acroperus, Camptocercus,* and *Chydorus. Alona* s.l. rivals *Daphnia* as the most species-rich genus of the order^9^. All *Alona* fossils seen so far had two connected head-pores (Fig. 2), placing them near or in the somewhat controversial genus *Biapertura*. In modern European faunas, and except for *Biapertura affinis,* three-pored species are dominant, while two-pored ones tend to be typical of warm-climate faunas. The postabdomen, another typical anomopod structure functioning more or less as a ‘tail’, is similar but not identical to that of *Biapertura affinis*. Furthermore, distinct other species (Fig. 2) have been found as well.

**Figure 3:**
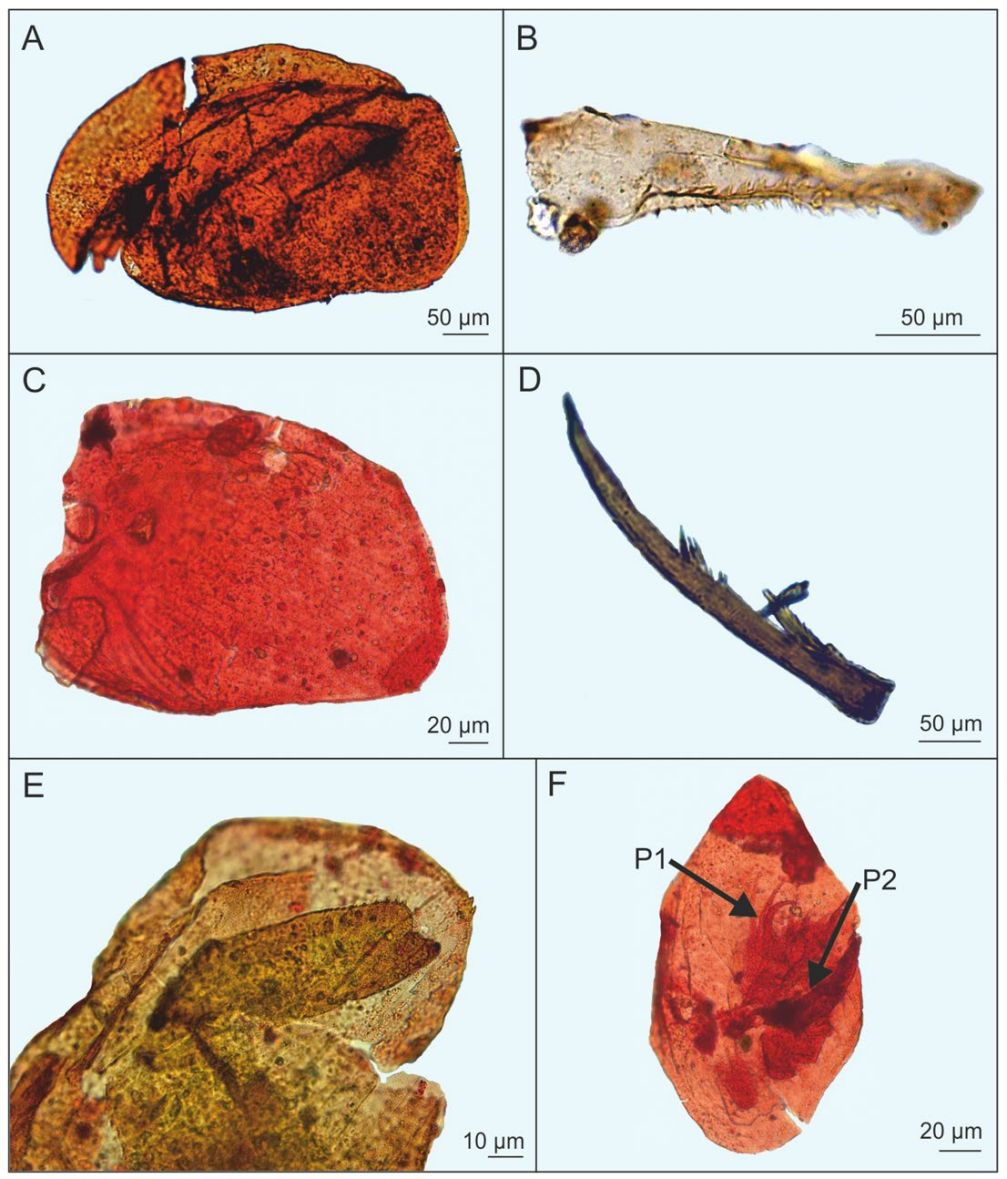
*Acroperus*-*Camptocercus* from Miocene mudstone at Bełchatów [(**A**) complete animal with shell, headshield, and first antenna; (**B**) abdomen; (**C**) shell; (**D**) claw; (**E**) shell with abdomen; (**F**) headshield with soft parts, viz. first and second trunk limbs with IDL setae conserved] (photo: A. Pociecha, E. Zawisza).

Another type of fossils (Fig. 3) shares characters of the genera *Acroperus* and *Camptocercus*. Some body parts suggest the presence of *Acroperus* cf. *harpae*, others are *Campocercus* like. One three-dimensional fossil of a first trunk limb (P1) with soft parts preserved shows an IDL (inner distal lobe) with two setae and a moderately well developed claw. In *Acroperus,* a seta instead of a claw is found here. In *Camptocercus*, P1 has a big hook here, and our fossils are therefore intermediate between both genera, suggesting the fossil to belong to an extinct ancestral stage. The genus *Chydorus* is represented by *C. sphaericus*-like fossils (Fig. 3), but the postabdomen is needed to decide on its taxonomic status.

**Figure 4:**
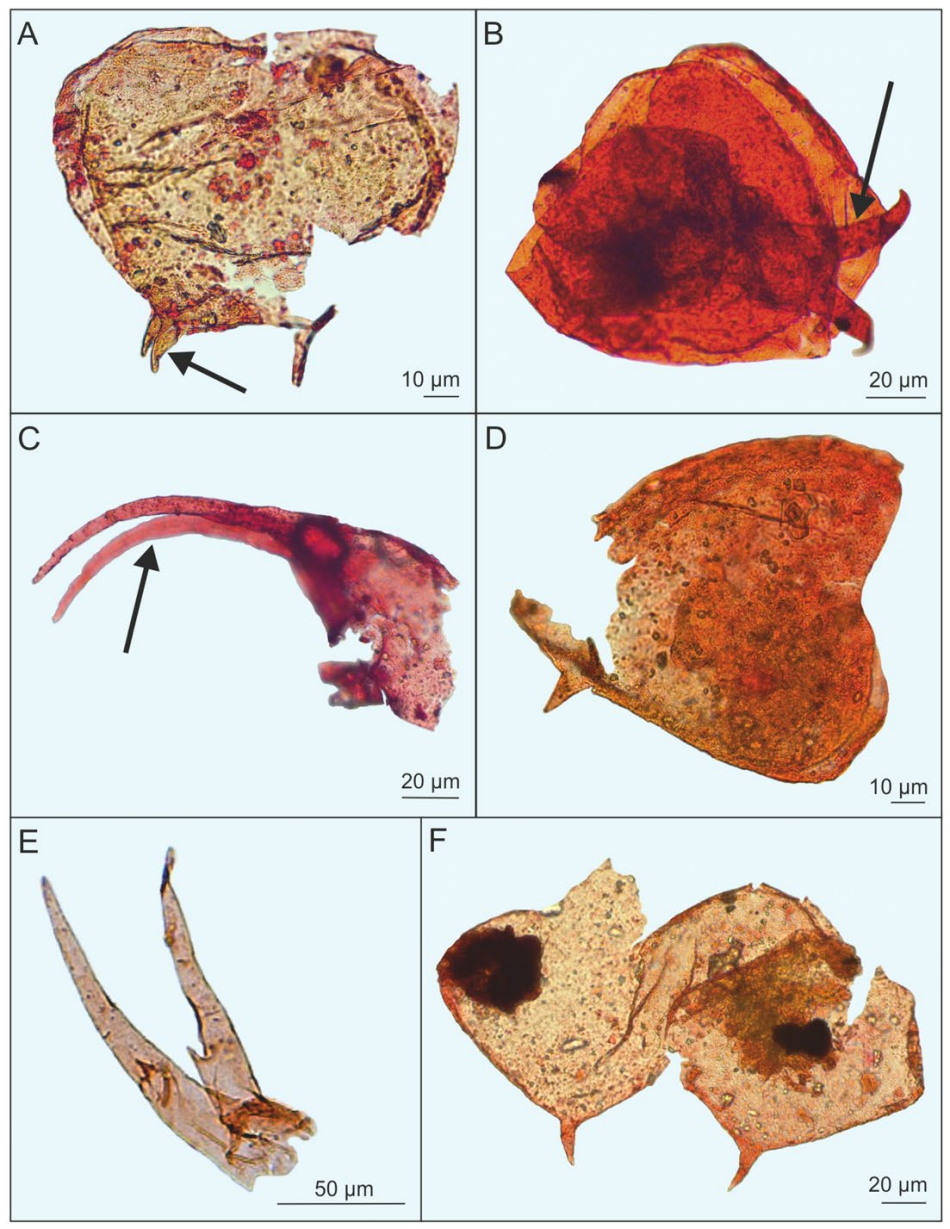
*Bosmina* from Miocene mudstone from Bełchatów [(**A**, **D**, **F**) shell; (**B**) shell with headshield; (**C**) headshield with first antenna; (**E**) first antenna)] (photo: A. Pociecha, E. Zawisza).

Significantly, Bosminidae in our material were almost as abundant as *Alona*, while in this family no pre-Pleistocene fossils were previously known at all. Most body parts have been recovered (Fig. 4), but not the postabdomen, which is diagnostic. There is strong variation in the fossils, like in the length of the first antenna (compare Fig. 4B with Fig. 4C,E) and the posterior mucro of the valve. It is impossible to decide whether these antennae belong to several or to a single cyclomorphotic species. However, the distinctly swollen mucros like in Fig. 4A, arrow, are not known in any modern *Bosmina,* and suggest an extinct taxon.

Daphniid fossil evidence goes back to the Jurassic/Cretaceous boundary. Fossils are fairly common but only consist of resting eggs (ephippia). Ephippia can tentatively be identified to genus level, which has led to the idea that the two subfamilies of Daphniidae have been around, apparently unchanged, for more than 140 million years^9^. This is known as the morphological stasis hypothesis. Its factual basis is weak although we accept that true *Daphnia* ephippia occur in the cretaceous of China, Mongolia and Australia^9^. Credible ephippia have also been isolated from Eocene age Messel pit-oil shales^11^. *Moina* (a water flea related to Daphnia) ephippia have been recovered from the early Miocene Barstow formation^12–14^. In the latter place, fossils are three-dimensional, like in our case in Bełchatów. It should be emphasized, however, that at Bełchatów no daphniid fossils have to date been discovered.

A variety of plant and animal groups, including water plants like *Potamogeton* have been documented from the Bełchatów site. Many are as beautifully preserved as the cladocerans^3–7^ and attempts have been made to reconstruct the climatic environment in which they lived. It is suggested to have been warmer than today (average 13.5°–16.5°C, cooling down gradually towards the end of the Miocene). The water was probably slowly flowing, through what may have been a swamp or a group of oxbow lakes from freshwater ecosystems^2,15^.

That we recovered no daphniid fossils may refine our insight into the nature of the Bełchatów aquatic environment: We favour the idea of a series of shallow oxbow lakes in a climate warmer than todays, with at least locally an abundance of macrophytes. Chydorids thrive in such environments, and the genus *Simocephalus* among daphniids also prefers such an environment. *Simocephalus* is quite common and widespread in modern weedy lakes but it has not been found in Bełchatów either. Like Daphnia, Bosminids are truly planktonic and need open water. Therefore, the Bełchatów lakes were probably a patchwork of macrophyte beds and open water.

So where did all the daphniids go? Daphniids are also relatively large species (up to several mm in size), while chydorids and bosminids are typically below a millimeter in size. This suggests size-selective predation on the zooplankton, most probably by fish^16^. Fish (tench, pike, and unidentified species) were present at Bełchatów^4^, at least until the middle Miocene. Tench is an omnivore and Pike is zooplanktivorous in its early stages, turning to piscivory in later life. Predation-driven exclusion of these large cladocerans may therefore well have acted in concert with the physical environment.

Predatory exclusion is seldom absolute, especially in a non-tropical climate with a cold season (‘winter’) during which predation is relaxed. This allows the prey to recover. In Bełchatów, we see no such a recovery, so another factor must have intervened. We suggest this may have been linked to water chemistry. Cladocerans, though of worldwide occurrence, are sensitive to water chemistry, more than the copepods, the second main group making up the zooplankton. We are unable to specify the precise nature behind the chemical exclusion of daphniids, but there are many examples of lakes in which Cladocera are chemically depressed. Sometimes, like in Lake Tanganyika (Africa), and the Mallili lakes (Indonesia), Cladocera are excluded altogether^17,18^.

In conclusion, our Miocene assemblage significantly expand our insights in anomopod evolution. They resemble modern faunas but at the same time show stronger signs of evolution at the species and at the genus level than expected under the morphological stasis hypothesis based on Daphnia ephippia. We also find clear indications of a species diversity that resembles todays’, with morphologies that look familiar at first sight but prove divergent in the details.

Bosminid fossils used to be known from the Pleistocene only, but in the Bełchatów fauna they are remarkably abundant and some of them show an unfamiliar morphology. This is best interpreted as a case of paleo-competitive release. Daphniids normally dominate the pelagic and keep bosminid abundances down. But their absence gives these small crustaceans a chance to prosper and dominate the open waters.

## Methods

Cladoceran remains were first found during palynological analysis of samples from a collection encoded KRAM-P 225. Only recently, the material containing cladocerans was examined in detail.

The fossils include exoskeletons or almost complete animals, and are encased in mudstone from which they can be isolated by gentle preparation^19^. Around 50 gr of mudstone was incubated with a 10% solution of sodium hexametaphosphate and was allowed to macerate overnight under gentle heating. After washing with distilled water, the sample was heated with 10% KOH for twenty minutes, washed on a 30 μm sieve, and stained with safranin. We applied no stirring, not to dislocate the delicate fossils. Some showed almost intact soft parts, such as trunk limbs and mouth parts (the labrum). They were, in fact, better preserved than most Holocene (sub)fossils. Often, the fossils were three-dimensional with well preserved soft parts.
